# Differential induction of total IgE by two *Salmonella enterica* serotypes

**DOI:** 10.3389/fcimb.2015.00043

**Published:** 2015-05-26

**Authors:** Zhanna A. Ktsoyan, Mkhitar S. Mkrtchyan, Magdalina K. Zakharyan, Armine A. Mnatsakanyan, Karine A. Arakelova, Zaruhi U. Gevorgyan, Lusntag A. Ktsoyan, Anahit Ì. Sedrakyan, Alvard I. Hovhannisyan, Karine A. Ghazaryan1, Anna S. Boyajyan, Rustam I. Aminov

**Affiliations:** ^1^Institute of Molecular Biology of National Academy of Sciences of Republic of ArmeniaYerevan, Armenia; ^2^“Nork” Clinical Hospital of Infectious Diseases, Ministry of Health of the Republic of ArmeniaYerevan, Armenia; ^3^Yerevan State Medical University after Mkthitar HeratsiYerevan, Armenia; ^4^National Veterinary Institute, Technical University of DenmarkFrederiksberg, Denmark

**Keywords:** *Salmonella*, salmonellosis, serotype, IgE, IL-17

## Abstract

The main goal of this study was to establish how the inflammation caused by infection with two different *Salmonella enterica* serotypes, *S*. Typhimurium and *S*. Enteritidis, may lead to the predisposition to allergy as measured by total IgE level in the blood. Infection by *S*. Typhimurium did not affect the systemic IgE concentration while in *S*. Enteritidis-infected patients there was a significant 3.5-fold increase. This effect was especially profound in patients >4 years old, with up to the 8-fold increase above the norm. The degree of dysbiosis in these two infections measured with the comparative counts of cultivated bacteria showed an inverse relationship with the IgE concentration. Earlier we reported the elevated level of IL-17 in patients infected by *S*. Enteritidis. In the current study a significant correlation was found between the concentrations of IL-17 and IgE suggesting a possible role played by this cytokine in triggering the production of IgE in response to *S*. Enteritidis infection.

## Introduction

Intestinal infectious diseases remain a serious problem for the health care systems. In many countries the most frequent intestinal infection is salmonellosis, which is caused by various serotypes of non-typhoidal *Salmonella* (Rabsch et al., 2001). Except for norovirus infections, *Salmonella* is a single most common causative agent among food-borne diseases (Centers for Disease Control and Prevention, 2013). Gastroenteritis is most frequently caused by *Salmonella enterica* serovar Typhimurium (*S*. Typhimurium) and *S. enterica* serovar Enteritidis (*S*. Enteritidis), which are also prevalent in Armenia (Ktsoyan et al., 2013). The former serovar has been predominantly reported before the 1980's but beginning from the 1990's the latter becomes the predominant serovar isolated from the salmonellosis cases (Angulo and Swerdlow, 1999; Munro et al., 1999; Tschape et al., 1999).

At the genomic level, *S. enterica* serovars are very close, with a large and stable core genome, while the accessory genome is dominated by mobile genetic elements such as phages, prophages, genomic islands, transposons and plasmids (Thomson et al., 2008; Jacobsen et al., 2011). Interestingly, a particular gene content often correlates with a serotype of *S. enterica* thus providing a genotypic background for the earlier circumstantial observations on the dependence of clinical picture of salmonellosis on the serotype (Jones et al., 2008).

The pairwise comparison of *S*. Typhimurium and *S*. Enteritidis similarly emphasizes the role of mobile genetic elements in genomic differences between the two. In particular, this is the presence of genes encoded by bacteriophages Fels-1, Fels-2, Gifsy-1, and Gifsy-2, as well as by two other phage-related regions in the chromosome of *S*. Enteritidis (Olson et al., 2007). Other differences include the lack SGI1 in *S*. Enteritidis as well as the carriage of dissimilar virulence plasmids by these two serovars. Despite a better understanding of genomic differences between the two serotypes, it remains unclear what factors contributed to the emergence of *S*. Enteritidis as a highly successful pathogen in the 1990's (Van Immerseel, 2010).

The normal microbiota plays a critical role in the establishment and maintenance of colonization resistance and proper immune responses. The impact on the intestinal microbiota in childhood is believed to be a major risk factor for chronic immune diseases such as allergy and autoimmune diseases in later life (Licciardi et al., 2010). During infection, *Salmonella* induces a strong pro-inflammatory response, which affects the normal intestinal microbiota and allows *Salmonella* to gain a competitive advantage (Stecher et al., 2007). The clearance of *Salmonella* infection, however, is not the end of the story. Gastroenteritis caused by *S*. Enteritidis during childhood, for example, is a risk factor for irritable bowel syndrome in adulthood (Cremon et al., 2014).

The etiology of allergic diseases remains uncertain, and many factors have been implicated as contributing to their development. One of the pioneering works in this area has been the hygiene hypothesis proposed by David Strachan (Strachan, 1989). The hypothesis implicated an insufficient microbial exposure in modern life that may result in the increasing number of allergic diseases. More recent works also suggested a major role played by commensal microbiota in the development of atopic disorders (Penders et al., 2007a,b). If the structure-and-function of commensal microbiota is compromised, this in turn may affect the proper functioning of the immune system and colonization resistance (Prakash et al., 2011). Because of the complexity of commensal microbiota, the molecular mechanisms, by which the microbiota as a whole exerts beneficial or detrimental effects, remain largely unknown (Sekirov et al., 2010).

The link between allergic disorders and infectious diseases, bacterial, and viral, remain contradictory. There are indications that early viral or bacterial exposure could be beneficial to inhibit excessive allergic Th2 reactions by inclining the immune system toward Th1 responses (Herz et al., 2000). It is known, for example, that attenuated *Salmonella* may reduce the induced airway inflammation and Th2 responses (Wu et al., 2006). At the same time, however, infections may worsen atopic conditions. For a long time the cause of allergic diseases has been predominantly discussed within the Th1/Th2 framework. Recently, however, a number of studies have implicated the Th17 pathway in asthma and allergic diseases (Oboki et al., 2008; Cosmi et al., 2011; Newcomb and Peebles, 2013; Manni et al., 2014; Naji et al., 2014). In summary, the prevailing views are largely within the context of alterations in the microbiome that interfere with immune system maturation (Feehley et al., 2012). The compounding factors for alterations are multiple and include decreased exposure to infections due to vaccination and sanitation, mode of delivery, antibiotic use, infant formulae, and diet.

Previously, we have reported that infection by two serotypes of *S. enterica* results in a serotype-specific host response, with the induction of a specific set of cytokines and antimicrobial protein calprotectin (Ktsoyan et al., 2013). In particular, the concentration of IL-1β was significantly higher in patients infected with *S*. Typhimurium while that of IL-17 was higher in patients infected with *S*. Enteritidis. On the basis of our previous findings, the main aim of this study was to identify factors that may play a role in the predisposition to allergy following infection by the two *S. enterica* serotypes.

The hallmark of allergic inflammatory response is the binding of IgE to an allergen (De Amici and Ciprandi, 2013). The level of systemic IgE is considered as a typical biomarker for allergies. Moreover, the level of total IgE may indicate the likelihood of sensitization even in patients with negative specific allergy tests (Kerkhof et al., 2003).

## Materials and methods

The study cohorts included patients with salmonellosis admitted to the infectious disease hospital Nork in Yerevan, RA. A total of 56 patients with acute salmonellosis caused by *S*. Typhimurium (η = 21) or *S*. Enteritidis (η = 35) and 18 healthy subjects were enrolled in this study. The effect of age was investigated by separating data from the above three cohorts into two age groups: (i) less than 4 years old (14 patients infected with *S*. Typhimurium, 15—with *S*. Enteritidis and 7 healthy subjects) and (ii) more than 4 years old (7 patients infected with *S*. Typhimurium, 20 with *S*. Enteritidis and 11 healthy subjects). Diagnosis was based on clinical presentation and laboratory analyses. Clinical picture consistent with gastroenteritis were: diarrhea, fever, nausea, vomiting, and abdominal cramps. Anamneses of the food consumed, water sources, social gatherings, any contact with a similar illness, and recent travels were also recorded. The patients or guardians reported no previous allergies or autoimmune diseases.

The patients selected for the study were not taking any type of medication, including antibiotics, before the hospital admission. Blood and fecal samples were taken on the first or second day of admission to the hospital. At the time of discharge from the hospital, no presence of *Salmonella* has been detected in the fecal samples or blood of any of the patients. For detoxification and rehydration, all patients were receiving the standard infusion therapy.

All study subjects (or parents or guardians if a child) gave their written consent to give fecal and blood samples for the study. The study protocol was approved by the Ethics Committee of the Institute of Molecular Biology NAS RA (IORG number 0003427, Assurance number FWA00015042, and IRB number 00004079).

The composition of intestinal microbiota was evaluated by standard microbiological methods and selective media using the fecal samples collected from patients with acute salmonellosis caused by *S*. Typhimurium or *S*. Enteritidis. The term “dysbiosis” is not an universally accepted clinical definition but is rather broadly defined as “… any change to the composition of resident commensal communities relative to the community found in healthy individuals” (Petersen and Round, 2014). We examined the degree of dysbiosis by counting the increase or decrease of certain fecal bacterial groups in salmonellosis compared to healthy control subjects. The degree of dysbiosis was determined on a 0–4 scale, ranging from 0 (normal) to 4 (severe dysbiosis) according to the previously proposed criteria (Mitrokhin et al., 1998; Tabolin et al., 1998).

Biochemical assays for identification of *Salmonella* were: fermentation of glucose, negative urease reaction, lysine decarboxylase, negative indole test, H_2_S production and fermentation of galactitol (dulcitol). Serotypes of *Salmonella* were determined using the standard Kauffman-White scheme with the use of commercially available polyvalent antisera for flagellar (H) and lipopolysaccharide (O) antigens.

The concentration of IgE in the sera of patients with acute salmonellosis due to *S*. Typhimurium or *S*. Enteritidis infection was used to estimate the predisposition to allergy. The IgE concentration was measured using the ECL technology. The corresponding concentrations were determined with the use of the immunoassay analyzer cobas e 411 (Roche, USA) according to the manufacturer's protocols. The concentration of IL-17 was measured as described before (Ktsoyan et al., 2013).

GraphPad Prism 5 (GraphPad Software, USA) was used to perform the Mann–Whitney *U*-test to determine the statistical significance of differences among the groups studied. The *p*-values < 0.05 were considered statistically significant. Discriminant function analysis was carried out with IBM SPSS Statistics 19 (IBM, USA).

## Results

### Systemic IgE in *S. Typhimurium* and *S. Enteritidis* infections

**Figure 1:**
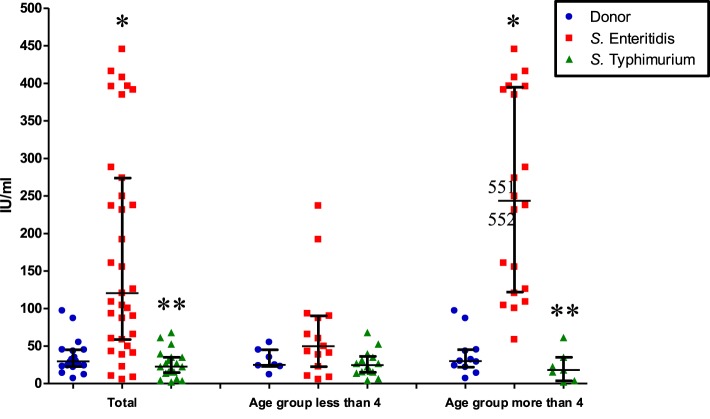
**Systemic IgE concentration in patients of different age and infected by different serotypes of *S. enterica* (median with interquartile range)**. Total: all patients infected by *S*. Typhimurium or *S*. Enteritidis, irrespective of age; control and either *S*. Enteritidis- or *S*. Typhimurium-infected. The second and third columns: patients are separated according to age and *S. enterica* serotypes. ^*^Significant difference compared to control (*p* ≤ 0.0002, Mann–Whitney *U*-test). ^**^Significant difference between *S*. Enteritidis- and *S*. Typhimurium-infected patients (*p* ≤ 0.0002, Mann–Whitney *U*-test).

We found a significant 3.5-fold increase of systemic IgE in patients of both age groups infected by *S*. Enteritidis compared to control (*p* < 0.0001), while in patients of both age groups infected by *S*. Typhimurium the level was approximately the same as in the control group (*p* = 0.12) (Figure 1). The level of IgE was 6-fold higher in patients of both age groups infected with *S*. Enteritidis compared to patients of both age groups infected with *S*. Typhimurium (*p* < 0.0001) (Figure 1). Compared to the healthy cohorts, *S*. Enteritidis-infected patients of the younger age had the IgE concentration 2-fold higher (*p* = 0.2, statistically not significant), while the elder patients with *S*. Enteritidis infection—8-fold higher (*p* < 0.0001). We observed a higher variability of IgE concentrationins in patients less than 4 years old infected by *S*. Enteritidis, which possibly compromised the statistical significance (Figure 1). In the elder age group the difference was consistent and significant, with the 86% of cases having the concentration of IgE higher than in healthy controls.

### Dysbiosis due to *S. Typhimurium* and *S. Enteritidis* infections

**Figure 2:**
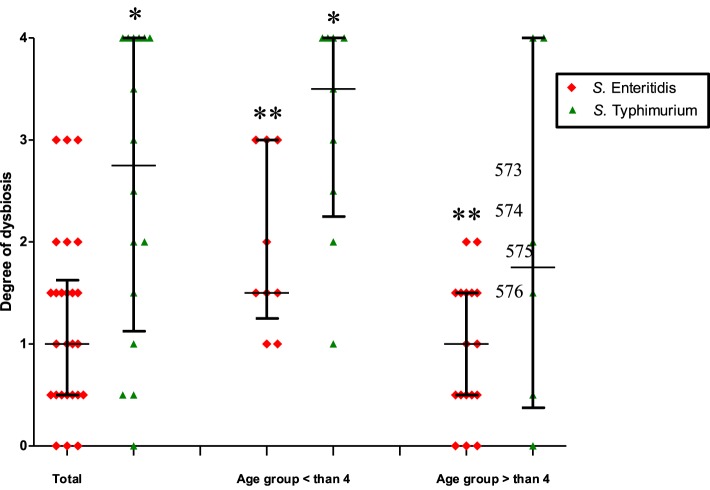
**The degree of dysbiosis in patients infected by *S*. Typhimurium and *S*. Enteritidis (median with interquartile range)**. Total: all patients infected by *S*. Typhimurium or *S*. Enteritidis, irrespective of age; the second and third columns: patients are separated according to age and *S. enterica* serotypes. ^*^Significant difference between *S*. Typhimurium- and *S*. Enteritidis-infected patients (*p* ≤ 0.05, Mann–Whitney *U*-test); ^**^Significant difference between two age groups of patients infected with *S*. Enteritidis (*p* = 0.01, Mann–Whitney *U*-test).

In the search for factors contributing to the elevated production of IgE due to *S*. Enteritidis infection we investigated a possible role of dysbiosis induced by these two serotypes of *S. enterica* (Table 1). Changes in the qualitative and quantitative composition of the fecal microbiota during the acute disease stage were investigated using the standard cultivation-based techniques with selective media. In general, the *Salmonella*-induced dysbiosis was characterized by a sharp decline in the numbers of *Bifidobacterium* spp., *Lactobacillus* spp., and *Escherichia coli* (Table 1). Also a quantitative increase in numbers of lactose-negative bacteria, *Enterococcus* spp.*, Candida* spp., and enteric bacteria with pathogenic potential was observed in fecal samples of *Salmonella*-infected patients compared to control. *E. coli* strains with low β-galactosidase activity were detected only in *S*. Enteritidis-infected patients while the increase in the lactose-negative *Enterobacteriacea* was more pronounced in patients infected with *S*. Typhimurium. The departure from the normal composition of gut microbiota was more pronounced in patients infected with *S*. Typhimurium compared to *S*. Enteritidis-infected. The degree of dysbiosis in the former group was significantly higher than in the latter (2.75 vs. 1.0, *p* = 0.005; Figure 2).

**Table 1:** **Dysbiotic changes due to *S*. Typhimurium and *S*. Enteritidis infections**.

**Microbial groups**		***S*. Typhimurium (% of patients^a^)**	***S*. Enteritidis (% of patients)**
1	Total *Salmonella* spp. count	↑ ^x^83.0^*^	↑ ^x^37.5^*^
2	Commensal *E. coli*	↓^y^55.0^*^	↓ ^y^25.5^*^
3	*E. coli* with low β-galactosidase activity	0	↑ ^x^10.5
4	Lactose-negative *Enterobacteriaceae*	↑ ^x^76.6^*^	↑ ^x^22.5^*^
5	Hemolytic *E. coli*	↑ ^z^30	↑ ^z^22.5^*^
6	Cocci among the total bacterial count (%)	↑ ^x^43.3	↑ ^x^47.5
7	Hemolytic *Staphylococcus* spp.	0	0
8	*Bifidobacterium* spp.	↓ ^w^80.7^*^	↓ ^w^52.5^*^
9	*Lactobacillus* spp.	↓ ^w^ 81.7^*^	↓ ^w^ 70.0^*^
10	*Enterococcus* spp.	↑ ^w^ 30.7	↑ ^w^ 28.0
11	*Proteus* spp.	0	0
12	*Staphylococcus aureus*	0	0
13	*Candida* spp.	↑ ^w^ 43.3^*^	↑ ^w^ 30^*^
14	Anaerobic cocci	0	0
%15	*Clostridium* spp.	0	0

a *Percentage of patients that have the increased (↑) or decreased (↓) count of the corresponding microbial groups compared to control*.

* *Statistically significant difference between the infected and control groups (p < 0.05)*.

x *Difference between the infected and control groups is ≥2-fold*.

y *Absence in the infected group*.

z *Presence in the infected group*.

w *Difference between the infected and control groups is ≥100-fold*.

Further intergroup and intragroup comparative analyses revealed that the degree of dysbiosis was most pronounced in the age group < 4 years old, with a higher average degree of dysbiosis in patients infected by *S*. Typhimurium compared to *S*. Enteritidis-infected (3.5 vs. 1.5, *p* = 0.03). In the age group >4 years old the difference was not significant (Figure 2). The intra-group analysis revealed a significant difference in the degree of dysbiosis between the <4 and >4 years old groups in the case of *S*. Enteritidis infections (1.5 vs. 1.0, *p* = 0.01) while in the case of *S*. Typhimurium infections the difference was not statistically significant (3.5 vs. 1.75, *p* = 0.22) (Figure 2). Thus, the comparative analyses demonstrated significant differences in the dysbiotic changes depending on the age of patients and the serotype of *S. enterica* that caused salmonellosis. The degree of dysbiosis in *S*. Enteritidis-infected patients, however, was significantly lower compared to *S*. Typhimurium infection. The cumulative dysbiotic score, therefore, could not be considered as a main factor contributing to the increased production of IgE in *S*. Enteritidis-infected patients.

### Multivariate discriminant function analyses

**Figure 3:**
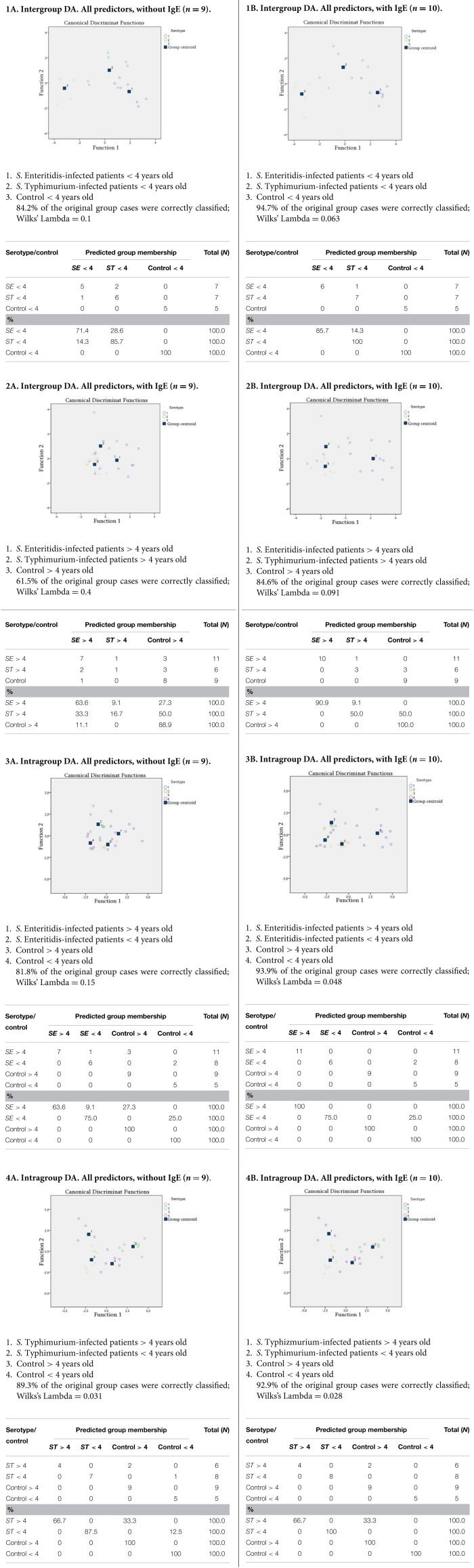
**DA models generated with predictor variables: counts of (i) *Salmonella*, (ii) *E. coli*, (iii) lactose-negative *Enterobacteriaceae*, (iv) *Bifidobacterium* spp., (v) *Lactobacillus* spp., (vi) *Enterococcus* spp., and (vii) *Candida* spp.; as well as (viii) the degree of dysbiosis, (ix) age ofsubjects, and (x) IgE concentration**. IgE concentration is omitted in models in column A and included in column B models.

To get into the finer level of resolution and establish the role of other possible factors involved, the raw data were further subjected to multivariate analyses to identify the relationships between more variables and their relevance to the correct classification of subjects in the study. In particular, we used discriminant function analyses (DA) with the aim to determine the distribution of patterns from the complex datasets obtained for each group studied. Various DA models have been tested, with the division into groups based on the age (patients and healthy subjects < or > than 4 years old) as well as on the *S. enterica* serotypes. The variable predictors used in modeling were the titers of certain microbial groups, which demonstrated significant changes compared to control (Table 1). Additional variable predictors included the degree of dysbiosis and age of the subjects thus making the number of variable predictors 9 (Figure 3A). Another predictor, concentration of IgE, was added to set of DA models to estimate its role in discrimination and correct classification (Figure 3B). The most successful models generated are summarized in Figure 3.

The DA analyses revealed a significant dependence of the dysbiosis on the serotype of *S. eneterica* in the younger age group (Figure 3.1A), while in the older age group this dependence was less evident (Figure 3.2A). In *S*. Typhimurium infections there was a significant dependence of dysbiosis from the age (Figure 3.4A), while this was less evident for the *S*. Enteriditis-infected patients (Figure 3.3A).

The results demonstrated that the DA models with the inclusion of a IgE concentration variable had a better predictive accuracy allowing a highly confident classification of subjects in the study (Figures 3.1B–3.4B). These results also suggest that the serotype of *S. eneterica* plays a role not only in the induction of a specific pro-inflammatory response (Ktsoyan et al., 2013) but is also involved in the formation of immunoglobulin responses in the adaptive arm of immunity that includes IgE production by B cells.

### Correlation between IL-17 and IgE in *S. Enteritidis*-infected patients

Previously we have revealed the serotype-specific induction of cytokines upon infection by *S*. Typhimurium and *S*. Enteritidis (Ktsoyan et al., 2013). In particular, acute infection by the latter serovar resulted in a significant increase of systemic IL-17 (6-fold increase compared to control and 2-fold increase compared to *S*. Typhimurium). Importantly, the elevated IL-17 level persisted in convalescent patients postinfection with *S*. Enteritidis (Ktsoyan et al., 2013).

**Figure 4:**
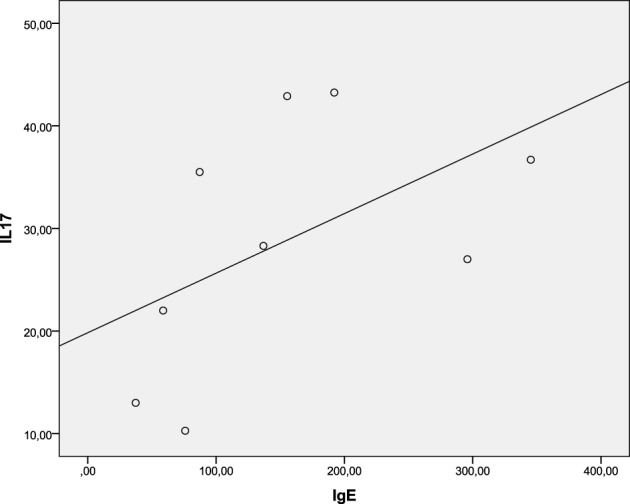
**Spearman's rank correlation between systemic IL-17 and IgE concentrations in *S*. Enteritidis-infected patients**. Spearman's rho = 0.683 [significant at the 0.05 level (2-tailed)].

Besides the involvement in a variety of host defenses and autoimmune diseases this cytokine has been recently implicated in a number of allergic diseases and asthma as well (Oboki et al., 2008; Cosmi et al., 2011; Newcomb and Peebles, 2013; Manni et al., 2014; Naji et al., 2014). In particular, it has been shown that IL-17 directly promotes IgE production in human B cells (Milovanovic et al., 2010). Thus we measured the systemic concentrations of IL-17 and IgE in nine patients infected with *S*. Enteritidis and performed statistical dependence analysis between the two variables (Figure 4). The Spearman's rho, 0.683, suggested a statistically significant dependence between the systemic concentrations of IL-17 and IgE in these patients. Need to reiterate here that the concentration of IgE is total, not specific, and it was measured, together with IL-17, on the first or second day of hospital admission. Thus this is not a textbook case of the conventional specific antibody response that needs the development of a sufficient Th cell response to promote the class switch, which requires about 8 days (Murray et al., 2012).

## Discussion

In this work we attempted to establish the impact of salmonellosis on the possible development of predisposition to allergies. For this, we monitored the systemic level of IgE, which is widely used as a marker for screening and detection of allergic conditions, in salmonellosis patients and control subjects. We found a significantly elevated level of IgE in the sera of patients infected by *S*. Enteritidis while infections caused by *S*. Typhimurium had no such effect. The impact of the *S*. Enteritidis infection on the elevated IgE concentration was more pronounced in the group of patients more than 4 years old while the younger patients demonstrated unsignificant departure from the control levels, possibly due to the extreme variability of IgE concentrations in this age group.

The main function of IgEs is providing immune defense against parasites such as helminthes and protozoa (Erb, 2007; Duarte et al., 2007). It was highly unexpected that infection by *S*. Enteritidis may result in the surge of systemic IgE, which production is primarily implemented via the Th2 signaling pathway (Shakib et al., 2008). On the contrary, it is generally accepted that bacteria such as *S*. Enteritidis induce mainly Th1-cell-dependent cellular and humoral responses, with a negligible contribution from the Th2 signaling pathway (Lehmann et al., 2006). This unusual finding led us to investigate the possible factors contributing to a significant induction of IgE in our patients infected by *S*. Enteritidis.

As noticed before, the host microbiota plays a substantial role in the development and maintenance of normal immune responses. Starting from the pioneering work of Strachan (1989), allergic conditions are mainly considered as a consequence of insufficient exposure of the immune system to the environmental microbiota in the contemporary lifestyle. There is a substantial body of evidence suggesting a direct link between allergic diseases and dysbiotic changes in gut microbiota (Prakash et al., 2011; Penders et al., 2007a,b).

Although the time-scale for the development of predisposition to allergic diseases is substantially longer compared to the time-course of *S.enterica* infection, we attempted to clarify a temporal dysbiosis effect during salmonellosis on the systemic IgE concentration. It is well known that infection by *Salmonella* causes inflammation and dysbiotic changes in the intestinal microbiota. In particular, the growth of the Bacteroidetes and Firmicutes is suppressed, which allows the overgrowth of *Salmonella* and *Enterococcus* (Deatherage Kaiser et al., 2013). To estimate the role of the dysbiosis factor, we investigated the composition of fecal microbiota in salmonellosis caused by the two serotypes of *S. enterica* using cultivation-based techniques. Our results indicated a significantly higher degree of dysbiosis in the case of *S*. Typhimurium infection compared to *S*. Enteritidis. The level of dysbiosis was higher in *S*. Typhimurium-infected patients of the younger (<4 years old) age compared to the elder (>4 years old) patients, which is not surprising since the comprehensive colonization resistance mechanisms may be not completely functional in the younger age group (Lawley and Walker, 2013). In general, there was a negative association between the degree of dysbiosis and IgE concentration. Although the importance of a balanced gut microbiota for the proper development and functioning of the immune system is well established, in particular for the prevention of allergic disorders, the salmonellosis-induced dysbiosis may not have an immediate effect on the production of this immunoglobulin isotype. Thus the temporal dysbiosis during salmonellosis can be ruled out as an imminent factor contributing to the excessive production of systemic IgE during *S*. Enteritidis infection.

To identify factors that may contribute to this phenomenon, we performed DA analyses with the involvement of almost all variables collected during this study. These results demonstrated that the main factor affecting the correct classification is the serotype of *S. enterica*. In our previous work we established that *S*. Enteritidis infection induces a significantly higher level of IL-17 compared to *S*. Typhimurium (Ktsoyan et al., 2013). Moreover, even after the clearance of *S*. Enteritidis infection, the post-infection convalescent patients still maintained an elevated systemic level of IL-17 (3-fold higher compared to control). It becomes increasingly clear that the Th17 lineage plays an important role in the host defense against a variety of pathogens and fills a crucial gap that could not be attributed to the previously defined functions of the Th1 and Th2 lineages (Curtis and Way, 2009). Besides the participation in the host response against pathogens, IL-17 is also involved in a number of other pathologies that include allergic and autoimmune diseases (Oboki et al., 2008; Cosmi et al., 2011; Newcomb and Peebles, 2013; Zhao et al., 2013; Manni et al., 2014; Naji et al., 2014). The Th17 pathway, therefore, has been proposed as a potential target for the treatment of autoimmune and allergic disorders (Robinson et al., 2013).

We hypothesized, therefore, that the excessive production of IL-17 during *S*. Enteritidis infection might contribute to the increased level of IgE found in these patients. Molecular mechanisms of how IL-17 promotes the IgE production in human B cells have been recently revealed by Milovanovic et al. (2010). They established that IL-17 directly promotes IgE production and increases the number of IgE-producing B cells. In the rodent models of murine atopic dermatitis, it has been shown that IL-17 serves as an inducer for Th2 immune responses, including the induction of IgE (Nakajima et al., 2014). On the other hand, infection by *S*. Typhimurium is characterized by a lesser level of IL-17 but a higher level of IL-1β compared to *S*. Enteritidis infection (Ktsoyan et al., 2013). IL-1β, together with IL-18, is a central target for the activation of inflammatory response by various inflammosomes (Sollberger et al., 2014). This interleukin appeared to be crucial for the early Th17 cell differentiation (Chung et al., 2009), and indeed we observed the elevated level of IL-17 in *S*. Typhimurium-infected patients compared to control (Ktsoyan et al., 2013). This level, however, remained lower than during *S*. Enteritidis infection and did not affect the IgE level. In a murine model, the attenuated *S*. Typhimurium may reduce the allergen-induced inflammation and Th2 response (Wu et al., 2006) thus demonstrating a protective effect of this *S. enterica* serotype against allergic conditions.

The pioneering work of Mosmann et al. (1986) has laid a foundation for the functional division of T helper cells into the two main populations, Th1 and Th2. With the accumulating knowledge, however, it becomes increasingly difficult to fit the experimental observations into this simple dichotomy. Later the list has been extended to include Treg cells (Asseman et al., 1999), Th17 cells (Steinman, 2007), Th9 cells (Veldhoen et al., 2008), and Th22 cells (Duhen et al., 2009; Trifari et al., 2009). These divisions are conditional to a certain extent, and the differentiation and stability of the phenotypes of T helper cells depend on a variety of external and internal cues to respond appropriately toward them (Murphy and Stockinger, 2010). The present view on allergic sensitization suggests the involvement of all T cell types including Th9, Th17, Th22, and Treg, in addition to Th1 and Th2 (van Ree et al., 2014). The resulting cytokine profile, with the dominance of IL-4 and/or IL-13, switch B cells to IgE production. In this work we provided direct experimental evidence suggesting a significant correlation between the elevated systemic concentrations of IL-17 and IgE in *S*. Enteritidis infection.

But what are the mechanisms specific for *S*. Enteritidis but absent in *S*. Typhimurium, which make the former more virulent and possibly contribute to the enhanced immunogenicity leading to the induction of IL-17 and IgE? There are two lines of evidence implicating the fimbriae of *S*. Enteritidis in conferring such traits compared to *S*. Typhimurium. First, whole-genome comparisons of *S*. Enteritidis and *S*. Typhimurium revealed an extensive core genome with >90% of coding sequences shared (Thomson et al., 2008). Coding sequences present in *S*. Enteritidis but absent in *S*. Typhimurium LT2 are dominated by prophage-related elements, which however, are related and harbor the same genes as prophage found previously in other *S. enterica*. Among the 13 fimbrial clusters in *S*. Enteritidis, there is a novel cluster not found in S. Typhimurium, which have been termed *peg* (Thomson et al., 2008). The role of the Peg fimbriae, in colonization and virulence has been established in a number of model experiments. In a recent study, a library of 54,000 transposon mutants of *S*. Enteritidis was screened for mutations attenuating the colonization ability *in vivo* using the BALB/c mouse model and a microarray-based negative-selection screening (Silva et al., 2012). Genes and genomic islands that are not present in *S*. Typhimurium or in most other *Salmonella* serovars but contribute to the pathogenicity of *S*. Enteritidis included: a type I restriction/modification system (SEN4290 to SEN4292), the *peg* fimbrial operon (SEN2144A to SEN2145B), a putative pathogenicity island (SEN1970 to SEN1999), and a type VI secretion system remnant SEN1001 (Silva et al., 2012). In another study of *S*. Enteritidis-specific virulence genes the transposon mutants have been screened for the attenuated invasiveness in human and chicken cells (Shah et al., 2012). The *S*. Enteritidis genes that are absent in *S*. Typhimurium or in most other *Salmonella* serovars included *pegD*, SEN1152, SEN1393, and SEN1966. In a later study of the same group the *pegD* mutant was found to be defective in intestinal colonization of chickens (Addwebi et al., 2014). Thus the consistent involvement of the *peg* fimbrial proteins in the virulence of *S*. Enteritidis makes them the prime candidates for the possible involvement in induction of IL-17 and IgE by this serovar.

Recent statistical data suggest an extremely high number of conditions with an allergic component, which include allergies to food, drugs and insects, as well as general and skin allergies, allergic rhinitis and sinusitis (http://www.aaaai.org/about-the-aaaai/newsroom/allergy-statistics.aspx). The worldwide rise in the number of these diseases is continuing for more than 50 years now (World Allergy Organization, 2013). In our work we established that certain bacterial infections could make a contribution to this general trend of ever growing sensitization by stimulating total IgE production. What is interesting, though, is that even closely related bacteria within the same species may induce differential IgE levels. Thus the exposure to bacterial infections may have differential outcomes for the development and severity of allergic disorders (Herz et al., 2000). More research is needed to understand the properties *S. enterica* serotypes that are closely related but, nevertheless, induce differential cytokine and IgE responses.

### Conflict of interest statement

The authors declare that the research was conducted in the absence of any commercial or financial relationships that could be construed as a potential conflict of interest.
